# Clinical significance of ribosomal protein S15 expression in patients with colorectal cancer liver metastases

**DOI:** 10.1002/jhbp.12012

**Published:** 2024-06-04

**Authors:** Yoshihiro Sakano, Daijiro Matoba, Takehiro Noda, Shogo Kobayashi, Daisaku Yamada, Yoshito Tomimaru, Hidenori Takahashi, Mamoru Uemura, Yuichiro Doki, Hidetoshi Eguchi

**Affiliations:** ^1^ Department of Gastroenterological Surgery, Graduate School of Medicine Osaka University Osaka Japan

**Keywords:** apoptosis, colorectal cancer, liver metastasis, RPS15

## Abstract

**Background:**

Liver metastasis is the most frequently observed distant metastasis of colorectal cancer, and the residual liver recurrence rate after hepatic resection is still high. To explore the mechanism of liver metastasis to discover potential new treatments, we assessed the relationship between the expression of differentially expressed genes (DEGs) and prognosis in patients with colorectal cancer liver metastasis (CRLM).

**Methods:**

The gene expression dataset was extracted from The Cancer Genome Atlas and the Gene Expression Omnibus. Significance analysis of DEGs between tumor and normal samples of colorectum, liver, and lung was conducted. A total of 80 CRLM patients were studied to assess the expression of *RPS15*, characteristics, and outcomes. We examined the relationships of RPS15 expression to cell viability and apoptosis in vitro and vivo.

**Results:**

Significance analysis identified 33 DEGs. In our cohorts, the overall survival rates were significantly lower in the high‐RPS15‐expression group, and high expression of RPS15 was an independent and unfavorable prognostic factor in recurrence‐free survival and overall survival. Knockdown of RPS15 expression reduced the proliferative capacity of colorectal cancer cells and increased BAX‐induced apoptotic cell death.

**Conclusions:**

RPS15 expression is an independent prognostic factor for CRLM patients and might be a novel therapeutic target for CRLM.

## Graphical abstract

This report by Sakano and colleagues is the first to demonstrate that ribosomal protein S15 is a potential prognostic predictor and therapeutic target in patients with liver metastases from colorectal cancer. This conclusion was based on in vitro and in vivo studies using colorectal cancer cells and clinical specimens.

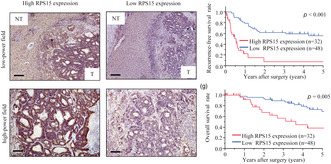

## 1 INTRODUCTION

Colorectal cancer (CRC) is one of the most common digestive tract malignancies worldwide, with high prevalence and mortality. Distant metastasis is the leading cause of cancer‐associated death in CRC patients.^1^ The 5‐year survival rate is 92% for patients with local disease, while it declines sharply to 53% and 11% for patients with regional and distant metastasis.^2^ Colorectal liver metastasis (CRLM) is one of the most prognostic factors in colorectal cancer, and surgery is the first choice if the primary tumor or other distant metastatic lesions are controlled and resectable.^3^, ^4^ However, the residual liver recurrence rate after hepatic resection is still high at about 70%, and the 5‐year survival rate after radical resection is about 40%.^5^, ^6^ There is therefore an urgent need to explore the mechanism of liver metastasis and seek a new strategy for CRLM treatment.

In many previous studies, the gene expression profiles of paired tumor and normal tissue samples from The Cancer Genome Atlas (TCGA) datasets were analyzed, and a number of genes that are significantly up‐ or downregulated in different types of cancer in comparison with their expression in normal tissue were identified.^7^ However, analysis of the primary cancer and normal tissue samples has not led to the development of drugs that can improve prognosis. Thus, some studies using samples from metastases, especially liver metastases from colorectal cancer, have been conducted in recent years.^8^

To explore the mechanism of liver metastasis, we assessed the relationship between the expression of differentially expressed genes (DEGs) from liver metastatic lesions, lung metastatic lesions, and primary lesions, using the public databases. We then identified the candidate gene set specific to liver metastasis and evaluated the clinical significance of the specific genes to explore effective therapeutic targets for liver metastases of colorectal cancer.

## 2 METHODS

### 2.1 Reagents

These materials included low‐glucose Dulbecco's modified Eagle's medium (DMEM), phosphate‐buffered salts, penicillin/streptomycin, ethylenediamine tetraacetic acid, fetal bovine serum (FBS), bovine serum albumin, and Tween 20. The materials used in this study were purchased from various reputable suppliers, including Nacalai Tesque (Kyoto, Japan), Gibco (Invitrogen, Life Technologies, Ghent, Belgium), Sigma‐Aldrich (Bornem, Belgium), and Fujifilm Wako Pure Chemical Corporation (Osaka, Japan).

### 2.2 Cancer cell lines and culture conditions

We used three human colorectal cancer cell lines (DLD‐1, SW480, HCT116) purchased from the American Type Culture Collection (Virginia, USA). Cells were maintained in low‐glucose DMEM supplemented with 10% FBS and cultivated in a humidified incubator at 37C with 5% CO_2_.

A highly liver metastatic colorectal cancer cell line (HCT116‐LM) was established as previously described.^9^ Briefly, a suspension of 10 × 10^5^ HCT116 cells was injected into the spleen of each of two mice in a volume of 100 μL. Three or 4 weeks after injection, liver metastatic tumors were removed, minced, and incubated with collagenase type I. The tumor cells were then injected back into the spleens of a second set of mice. This procedure was repeated for three cycles. The liver metastatic cells obtained from the HCT116 cells after three cycles were designated HCT116‐LM.

### 2.3 Capture gene expression profiles and clinical information from TCGA dataset and GEO database

RNA sequence data and corresponding clinical information on colon adenocarcinoma cases were obtained from TCGA via the Broad Institute's Firehose (http://firebrowse.org/?cohort=COAD, RNA‐seq, Illumina). This resource provides RNA‐seq data generated using Illumina technology, as previously described.^10^ Clinical information was available for 57 of the 457 COAD cases with mRNA expression profiles. Moreover, expression profiles were obtained for 57 paired noncancerous colon samples. Additionally, mRNA expression profiles of colorectal cancer were obtained from the Gene Expression Omnibus (GEO) database (GSE41258) (https://www.ncbi.nlm.nih.gov/geo/query/acc.cgi?acc=GPL11154). Expression profiles were also acquired for 186 primary colorectal cancer samples and 54 noncancerous colon samples, 47 liver metastasis samples and 13 noncancerous liver samples, and 20 lung metastasis samples and seven noncancerous lung samples. DEGs were identified for each of (1) primary tumors, (2) liver metastases, and (3) lung metastases based on fold change >1.5 and *p* < .05. (4) TCGA was used to identify the genes involved in overall survival (*p* ≤ .1). Next, we narrowed down the DEGs specific to liver metastases using (1)–(3) and identified genes common to (4) as candidate DEGs that contribute to survival. Using the median ribosomal protein S15 (RPS15) expression in the TCGA cohort as a cutoff value, we divided patients into two groups (a high‐RPS15‐expression group and a low‐RPS15‐expression group).

### 2.4 Clinical tissue samples

Clinical tissue samples were obtained from the Department of Gastroenterological Surgery, Osaka University Hospital, and included liver specimens from 80 patients who had undergone hepatectomy for CRLM and the specimens of primary lesions from 37 patients who had undergone surgery for primary colorectal cancer from January 2010 through December 2016. Classification of histology was performed by a pathologist according to the General Rules for the Clinical and Pathological Study of Colorectal Cancer in Japan, ninth edition. The human ethics review committee of the Graduate School of Medicine, Osaka University (20548) approved the use of resected specimens. All patients gave their written informed consent for the research use of their surgical specimens and clinicopathological data.

### 2.5 Immunohistochemistry

To assess RPS15 expression, immunohistochemical studies of clinical tissue samples were performed as previously described.^9^, ^11^ The formalin‐fixed, paraffin‐embedded tissues were subjected to deparaffinization and incubated with an RPS15‐specific antibody (anti‐RPS15 rabbit polyclonal antibody: Abcam, Cambridge, UK) overnight at 4°C. Biotin‐conjugated secondary antibodies (Vector Laboratories, Burlingame, California, USA) were utilized for antibody detection. The cytoplasmic RPS15 expression in cancer lesions in CRLM specimens was evaluated, and all sections were counterstained with hematoxylin. Those that stained more intensely than the positive control esophageal cancer sample were designated as the high‐expression group, and those that stained less intensely than the esophageal cancer sample were designated as the low‐expression group. All photographs were taken with a fluorescence microscope (BZ‐X700, Keyence, Osaka, Japan). The detection of apoptotic cell death was performed by the transferase‐mediated dUTP nick end labeling (TUNEL) technique using the MEBSTAIN apoptosis TUNEL kit Direct (MBL, Nagoya, Japan) as described previously.^10^ The counting of apoptotic tumor cells was performed by using the number of TUNEL‐positive nuclei.

### 2.6 Quantitative reverse transcription‐polymerase chain reaction

The method of quantitative reverse transcription‐polymerase chain reaction (qRT‐PCR) was carried out in accordance with a previously described protocol.^12^ In short, total RNA was isolated using TRIzol reagent (Invitrogen). Complementary DNA was synthesized with the reverse transcription system (Promega, Tokyo, Japan). Then, qRT‐PCR was performed using specifically designed oligonucleotide primers on an Applied Biosystems ViiA7 Real‐Time PCR System (Thermo Fisher Scientific, Massachusetts, USA). The quantification of amplification products was performed using Thunderbird SYBR qPCR Mix (Toyobo, Osaka, Japan). The expression levels of the target gene were normalized to those of β‐actin, which was used as an endogenous control.

### 2.7 Downregulation of RPS15 gene expression by small interfering RNA

To downregulate RPS15, colon cancer cell lines were transfected with 5 nM small interfering RNA (siRNA; ID s194765 and s194766, Life Technologies, Carlsbad, California, USA) using lipofectamine RNAiMAX reagent (Invitrogen) according to the manufacturer's instructions. The same method was performed with nontargeting siRNA (scramble siRNA, Silencer Select negative control #1 siRNA; Life Technologies) as a control.

### 2.8 Western blotting

Western blot analysis was conducted in accordance with the previously described protocol.^13^ Total protein (20 μg) was separated using 4%–15% SDS‐PAGE and then electrotransferred onto polyvinylidene difluoride membranes. The membranes were incubated for 1 h with several primary antibodies, including anti‐RPS15, anti‐cleaved caspase‐3 (Abcam), anti‐Bcl‐2‐associated X protein (Bax), anti‐caspase‐3 (Cell Signaling Technology, Massachusetts, USA), and anti‐β‐actin (Sigma‐Aldrich, St. Louis, USA). The membranes were incubated with HRP‐linked anti‐rabbit IgG (GE Healthcare Biosciences, Piscataway, New Jersey, USA) at room temperature for 1 h. The antigen–antibody complexes were then detected using an ECL prime western blotting detection kit (GE Healthcare Biosciences).

### 2.9 In vitro proliferation assays

Tumor cell viability was evaluated using a cell counting kit‐8 (CCK‐8) assay (Dozindo Laboratories, Kumamoto, Japan), as previously described.^9^ Each cancer cell line was seeded into a 96‐well plate (2 × 10^3^ cells/well) and incubated for 24 h. Following transfection with RPS15 siRNA or nontargeting siRNA, the CCK‐8 solution was added to each well at 0, 24, 48, 72, and 96 h. The cells were then incubated for 3 h, and the absorbance was measured using a microplate reader. The results are presented as the absorbance relative to 0 h of CCK‐8 exposure.

### 2.10 Flow cytometry

For the detection of apoptotic cells, Annexin V assays were performed as previously described.^10^ At 72 h after transfection with RPS15 siRNA and nontargeting siRNA, cells were stained with annexin V‐FITC and PE‐conjugated propidium iodide (PI; BioVision Research Products, Mountain View, California, USA) according to the manufacturer's instructions. The number of apoptotic cells, defined as those positive for annexin V or PI, was determined by counting approximately 10 000 cells using a FACS Canto II (BD Biosciences, Franklin Lakes, New Jersey, USA). The TUNEL technique was also used to detect apoptotic cell death using the MEBSTAIN apoptosis TUNEL kit direct (MBL, Nagoya, Japan) as described previously.^10^ Apoptotic cells were identified and quantified using a FACS Canto II (BD Biosciences) by analyzing cells positive for TUNEL. Each sample was tested with approximately 10 000 cells.

### 2.11 Animal studies

Animal experiments were conducted using 8‐week‐old male BALB/cAJcl‐nu/nu immunodeficient mice (CLEA Japan, Tokyo, Japan). For in vivo tumor formation, 5 × 10^6^ HCT116 cells were injected into the spleen using a 27G needle, and then the spleen was removed as described previously.^14^ Four weeks later, the mice were sacrificed. The metastatic liver tumors were harvested, minced, and then incubated with collagenase type I. The tumor cells were filtered through a 70‐μm cell strainer, placed in DMEM in a 10‐cm dish, incubated until they reached confluency, and then again injected into the spleens of mice. We repeated this procedure for three cycles. The original human colorectal cancer cell line was designated as HCT116, and the colorectal cancer cells obtained after three cycles were designated as HCT116‐LM.

All trials were conducted under the ethical guidelines of the Declaration of Helsinki, the Japanese Ethical Guidelines for Human Genome/Gene Analysis Research, and the Osaka University Ethical Guidelines for Medical and Health Research Involving Human Subjects.

### 2.12 Statistical analysis

The clinicopathological characteristics of the patients were compared using statistical tests appropriate for the data type. The recurrence‐free survival (RFS) and overall survival (OS) were analyzed using a well‐established statistical method, and differences between survival curves were compared using a commonly accepted test. To evaluate the potential risks associated with the prognostic variables, both univariate and multivariate analyses were conducted using a Cox model. The hazard ratio (HR) and 95% confidence interval (CI) were determined. In vivo data are presented as the mean ± standard error of individual mice from a representative experiment. Statistical analyses were performed using either JMP software version 15.0 (SAS Institute Inc., Cary, NC, USA) or R version 3.1.1 (Vienna, Austria; http://www.r‐project.org/).

## 3 RESULTS

### 3.1 Identification of differentially expressed genes in colorectal liver metastases

**FIGURE 1:**
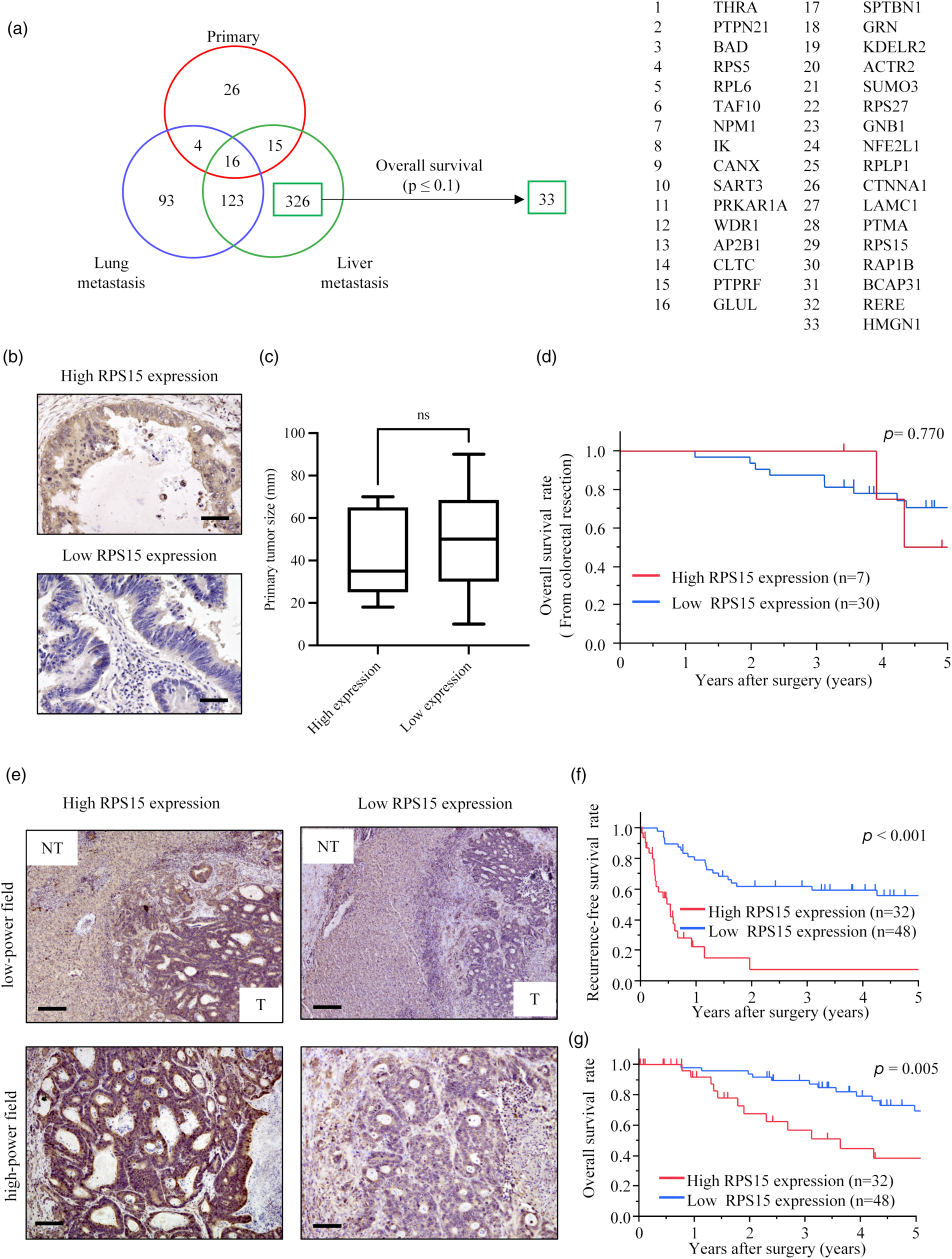
(a) The selection of differentially expressed genes in the Cancer Genome Atlas (TCGA) and the Gene Expression Omnibus (GEO) database. (b) Immunohistochemical analysis of RPS15 expression in primary colorectal cancer specimens. Upper panel: high RPS15 expression in primary colorectal cancer. Lower panel: low RPS15 expression in primary colorectal cancer. (c) The box plot of the correlation between primary tumor size and RPS15 expression. (d) Overall survival curves for colorectal cancer (CRC) patients after colectomy. (e) Immunohistochemical analysis of RPS15 expression in colorectal cancer liver metastasis (CRLM) specimens. Left upper panel: high RPS15 expression in CRLM in a low‐power field. Right upper panel: low RPS15 expression in CRLM in a low‐power field. Left lower panel: high expression in CRLM in a high‐power field. Right lower panel: low expression in CRLM in a high‐power field. (f) Recurrence‐free survival curves for CRLM patients after hepatectomy. (g) Overall survival curves for CRLM patients after hepatectomy. Scale bar, 50 μm.

By mRNA expression analysis, DEGs were identified for each of (1) primary tumors, (2) liver metastases, and (3) lung metastases based on fold change >1.5 and *p* < .05: for (1) 61 genes, for (2) 480 genes, and for (3) 236 genes. We also identified 3961 genes in TCGA that contribute to survival. We identified 326 DEGs as specific to liver metastases that were identified as DEGs of liver metastasis and not as DEGs of primary tumor and lung metastasis. The schema is shown in Figure 1a. In addition, we identified 33 genes among 326 DEGs specific to liver metastasis that influence overall survival (Table S1). Among the list of 33 genes, we excluded the previously reported genes related to colorectal cancer progression. Then, we found several ribosome‐related genes among 33 candidate genes and focused on ribosome‐related genes that regulate cell activity. Among the remaining ribosome‐related genes, we selected RPS15 for further investigation, as it plays the most important role in ribosome formation and activity.

### 3.2 Immunohistochemical analysis of RPS15 in primary lesions and metastatic liver lesions of colorectal cancer, and prognosis according to RPS15 expression

In the resected CRC and CRLM tissues, cytoplasmic RPS15 expression was observed in the cancer lesions of CRC and CRLM. Representative RPS15 expression in CRC cases is shown in Figure 1b. Immunohistochemical analysis revealed that seven patients had high RPS15 expression and the remaining 30 patients low RPS15 expression. In nontumorous lesions, specific RPS15 expression was little observed. We conducted a correlation analysis between primary CRC tumor size and RPS15 expression. However, the results indicated that there was no significant correlation (Figure 1c). The OS curves in the two groups are shown in Figure 1d, and the difference was not statistically significant (5‐year OS: 66.7% vs. 68.5%, *p* = .770). Furthermore, Representative RPS15 expression in CRLM cases is shown in Figure 1e. Immunohistochemical analysis showed that 32 patients had high RPS15 expression and the remaining 48 patients low RPS15 expression. Table S2 summarizes the clinicopathological features of the CRLM patients with high and low RPS15 expression, including age, sex, body mass index, the location of the primary tumor, T factor, N factor, stage, histological classification of the primary tumor, timing of liver metastasis, the presence of neoadjuvant chemotherapy, Child‐Pugh classification, carcinoembryonic antigen (CEA) levels, carbohydrate antigen 19‐9 (CA19‐9) levels, tumor number of CRLM, tumor size, type of hepatectomy, the presence of recurrence, recurrence location, death and cause of death. There were no factors that differed significantly between the CRLM patients with high and low RPS15 expression except the presence of recurrence in Table S2. The recurrence rate was significantly higher in the group with high RPS15 expression (69% vs. 38%, *p* = .006). The RFS and OS curves for the two groups after hepatectomy are shown in Figure 1f,g. Both RFS and OS rates were significantly lower in the high‐RPS15 group than in the low‐RPS15 group (5‐year RFS rate: 7.9% vs. 55.8%, *p* < .001; and 5‐year OS rate: 38.3% vs. 69.4%, *p* = .005).

Univariate analysis of RFS identified the following four factors as significant: time of metastasis (synchronous), N factor (N1, N2), stage (III, IV), and RPS15 expression (high). Multivariate analysis revealed that RPS15 expression was the only independent and significant factor (Table 1). In the univariate and multivariate analyses of OS, the values of CEA and RPS15 expression were identified as independently significant in Table 2.

**TABLE 1:** Univariate and multivariate analysis of recurrence factors after hepatectomy.

	Univariate			Multivariate		
	HR	95% CI	*p*‐value	HR	95% CI	*p*‐value
Age (years) (≧66/<66)	0.627	0.334–1.175	.145			
Gender (male/female)	1.086	0.562–2.098	.805			
BMI (kg/m^2^) (≧25/<25)	1.388	0.639–3.012	.407			
Primary tumor location colon/rectum	0.897	0.483–1.664	.730			
Depth of invasion (T4/T1–T3)	1.360	0.648–2.853	.416			
Lymph node metastasis (N1, N2/N0)	2.664	1.271–5.586	.010	1.935	0.738–5.072	.180
TNM Stage (III, IV/I, II)	3.608	1.285–10.132	.015	1.262	0.271–5.867	.767
Primary histological type (well, mod/poor)	2.898	0.699–12.008	.142			
Synchronous/heterochronic	2.590	1.319–5.088	.006	1.867	0.836–4.172	.128
Neoadjuvant chemotherapy (no/yes)	1.035	0.558–1.919	.913			
CEA [ng/mL] (≧6.5/<6.5)	1.652	0.890–3.068	.112			
CA19‐9 [U/mL] (≧11.3/<11.3)	1.156	0.566–2.359	.691			
Number of liver metastasis (multiple/single)	0.782	0.353–1.731	.544			
Size of liver metastasis [mm] (≧50/<50)	0.833	0.249–2.792	.768			
Type of hepatectomy (partial/others)	0.785	0.393–1.570	.494			
RPS15 expression (high/low)	5.965	3.063–11.616	<.001	5.585	2.848–10.951	<.001

Abbreviations: BMI, body mass index; CEA, carcinoembryonic antigen; CA19‐9, carbohydrate antigen 19‐9; CI, confidence interval; HR, hazard ratio.

**TABLE 2:** Univariate and multivariate analysis of prognosis factors after hepatectomy.

	Univariate			Multivariate		
	HR	95% CI	*p*‐value	HR	95% CI	*p*‐value
Age (years) (≧66/<66)	1.711	0.767–3.813	.189			
Gender (male/female)	2.061	0.771–5.508	.149			
BMI (kg/m^2^) (≧25/<25)	0.958	0.328–2.802	.938			
Primary tumor location colon/rectum	1.546	0.667–3.584	.310			
Depth of invasion (T4/T1–T3)	1.507	0.601–3.782	.382			
Lymph node metastasis (N1, N2/N0)	1.623	0.647–4.067	.302			
TNM Stage (III, IV/I, II)	2.072	0.620–6.930	.237			
Primary histological type (well, mod/poor)	2.569	0.347–19.037	.356			
Synchronous/heterochronic	1.634	0.705–3.788	.252			
Neoadjuvant chemotherapy (no/yes)	1.164	0.528–2.566	.706			
CEA [ng/mL] (≧6.5/<6.5)	3.277	1.365–7.865	.008	2.996	1.243–7.223	.015
CA19‐9 [U/mL] (≧11.3/<11.3)	1.515	0.567–4.049	.407			
Number of liver metastasis (multiple/single)	0.782	0.353–1.731	.544			
Size of liver metastasis [mm] (≧50/<50)	0.833	0.249–2.792	.768			
Type of hepatectomy (partial/others)	0.804	0.319–2.029	.645			
RPS15 expression (high/low)	2.958	1.340–6.529	.007	2.646	1.197–5.847	.016

Abbreviations: BMI, body mass index; CEA, carcinoembryonic antigen; CA19‐9, carbohydrate antigen 19‐9; CI, confidence interval; HR, hazard ratio.

### 3.3 Knockdown of RPS15 expression in colorectal cancer cell lines by siRNA reduced the proliferative capacity in vitro

**FIGURE 2:**
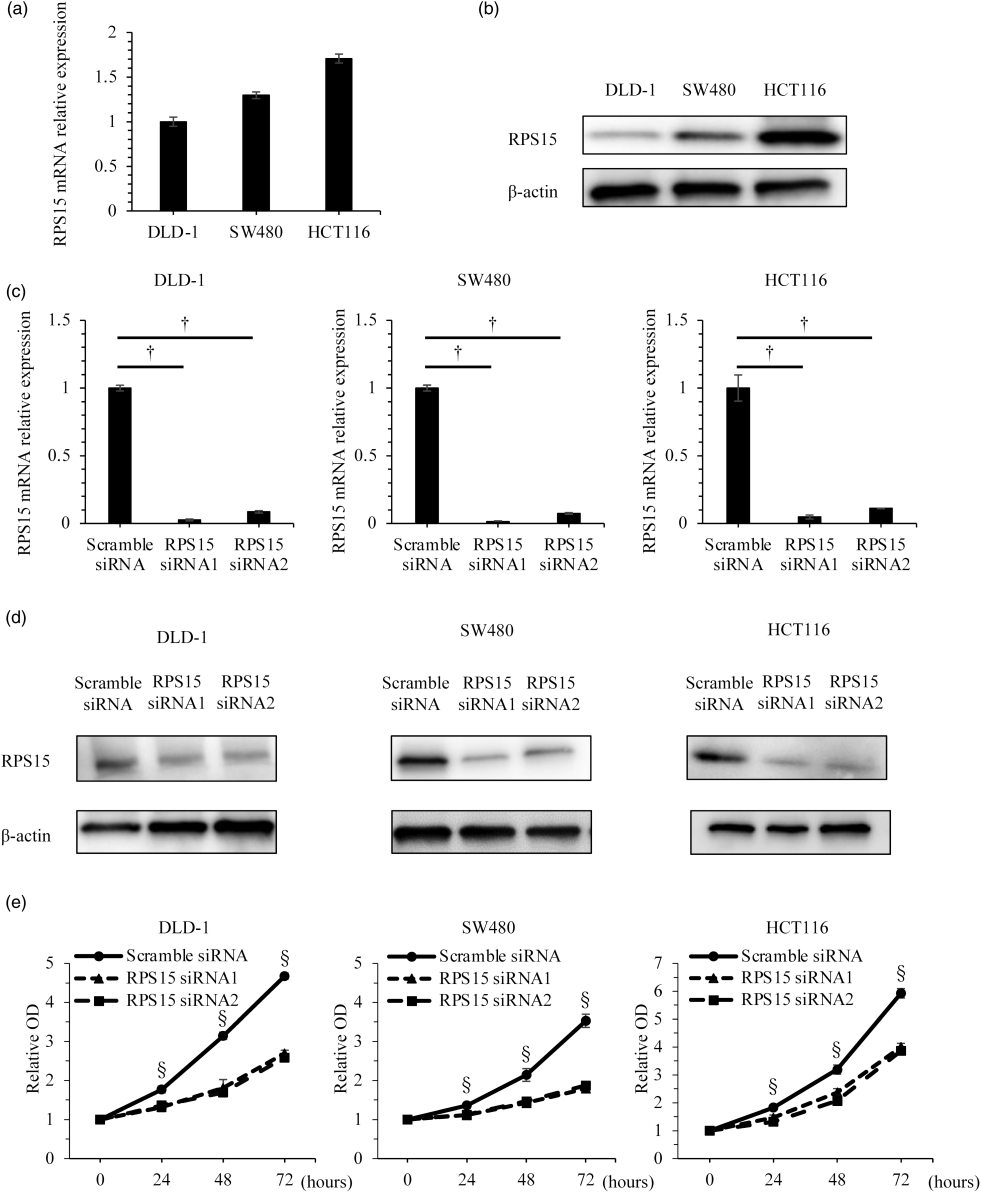
RPS15 knockdown in DLD‐1 cells, SW480 cells, and HCT116 cells using siRNA. Baseline RPS15 mRNA expression (a) and protein (b) in the three cell lines. Expression of RPS15 mRNA (c) and protein (d) after cell transfection with RPS15 siRNA or scramble siRNA. (e) Proliferation of DLD‐1 cells, SW480 cells, and HCT116 cells after RPS15 knockdown. ^†^ *p* < .01, ^§^ *p* < .001.

Three colon cancer cell lines (DLD‐1, SW40, and HCT116) were examined for RPS15 expression by qRT‐PCR. RPS15 expression in HCT116 was higher than it was in the other two cell lines (Figure 2a), and western blotting confirmed RPS15 expression in these cell lines (Figure 2b). When *RPS15* expression was knocked down with siRNA1 or siRNA2, *RPS15* mRNA relative expression was significantly suppressed to about 10% compared with the level in all three cell lines transfected with the scrambled siRNA (Figure 2c). Western blotting confirmed the suppression of RPS15 protein production in the three cell lines (Figure 2d). Proliferation was significantly inhibited in the RPS15 siRNA‐transfected cells to approximately 30%–50% of that in the scrambled siRNA‐transfected cells (Figure 2e).

### 3.4 Inhibition of RPS15 expression induces cancer cell apoptosis via BAX upregulation

**FIGURE 3:**
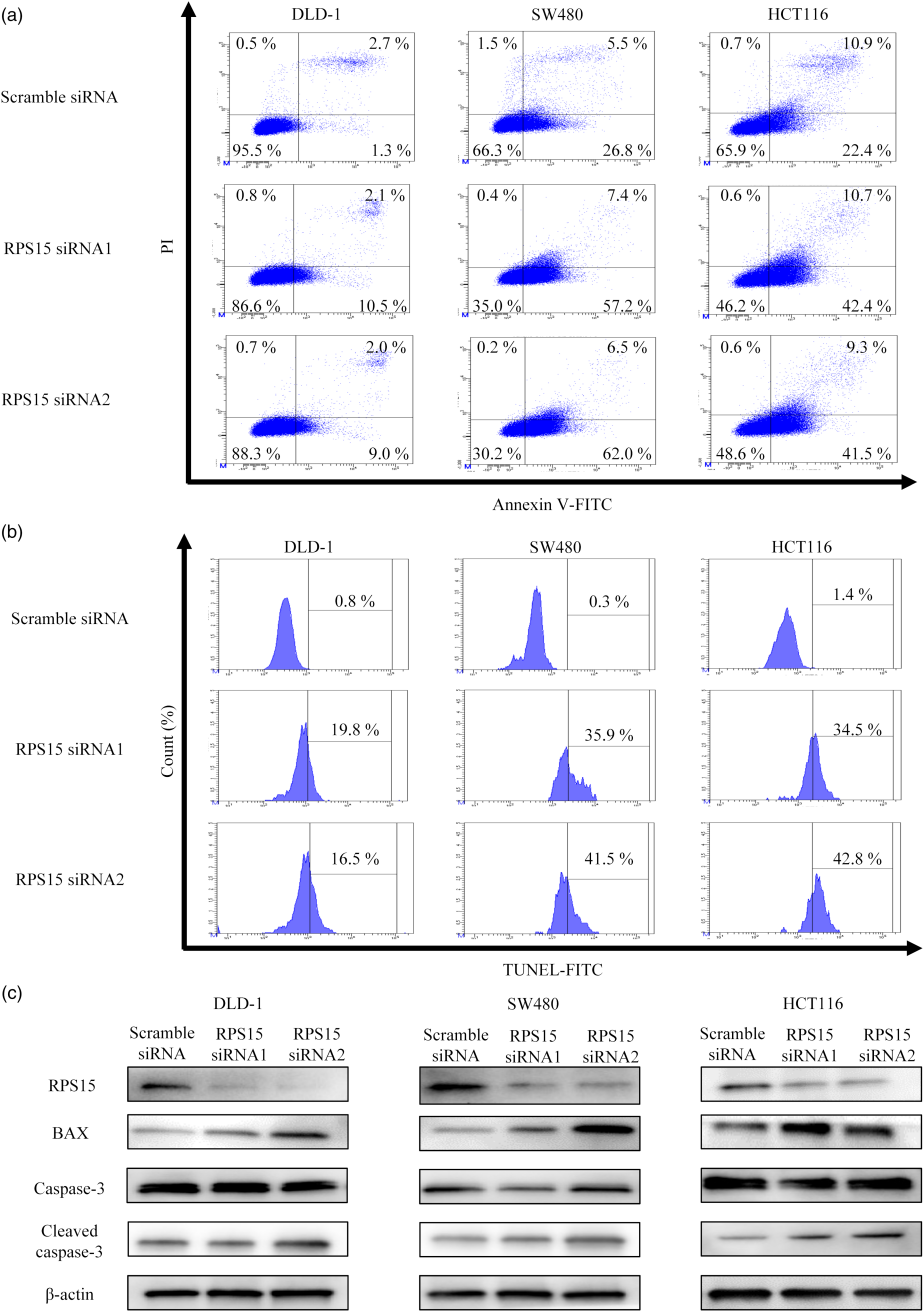
Downregulation of RPS15 induced apoptosis in vitro. (a) Annexin V assay after cell transfection with RPS15 siRNA or scramble siRNA in three cell lines. (b) TUNEL assay after RPS15 knockdown in three cell lines. (c) RPS15 and apoptosis protein expression after RPS15 knockdown in three cell lines.

To investigate the molecular mechanism underlying the effect of suppressing RPS15 expression, annexin V assays were performed to detect any change in cancer cell apoptosis. Knockdown of RPS15 increased cell apoptosis compared with scrambled siRNA‐treated control cell apoptosis in all three cancer cell lines (Figure 3a). Furthermore, TUNEL assays showed that knocking down RPS15 with siRNA1 or siRNA2 increased TUNEL positivity in the three cell lines to 17%–43% of the cells (Figure 3b). The silencing of RPS15 expression by the transfection of RPS15 siRNA1 or siRNA2 increased the expression of the apoptosis proteins BAX and cleaved caspase‐3 (Figure 3c). Thus, RPS15 expression could be involved in apoptosis protein expression via BAX suppression.

### 3.5 A highly liver‐metastatic cell line (HCT116‐LM) established by in vivo selection

After three cycles, a highly liver‐metastatic cell line (HCT116‐LM) was established.

**FIGURE 4:**
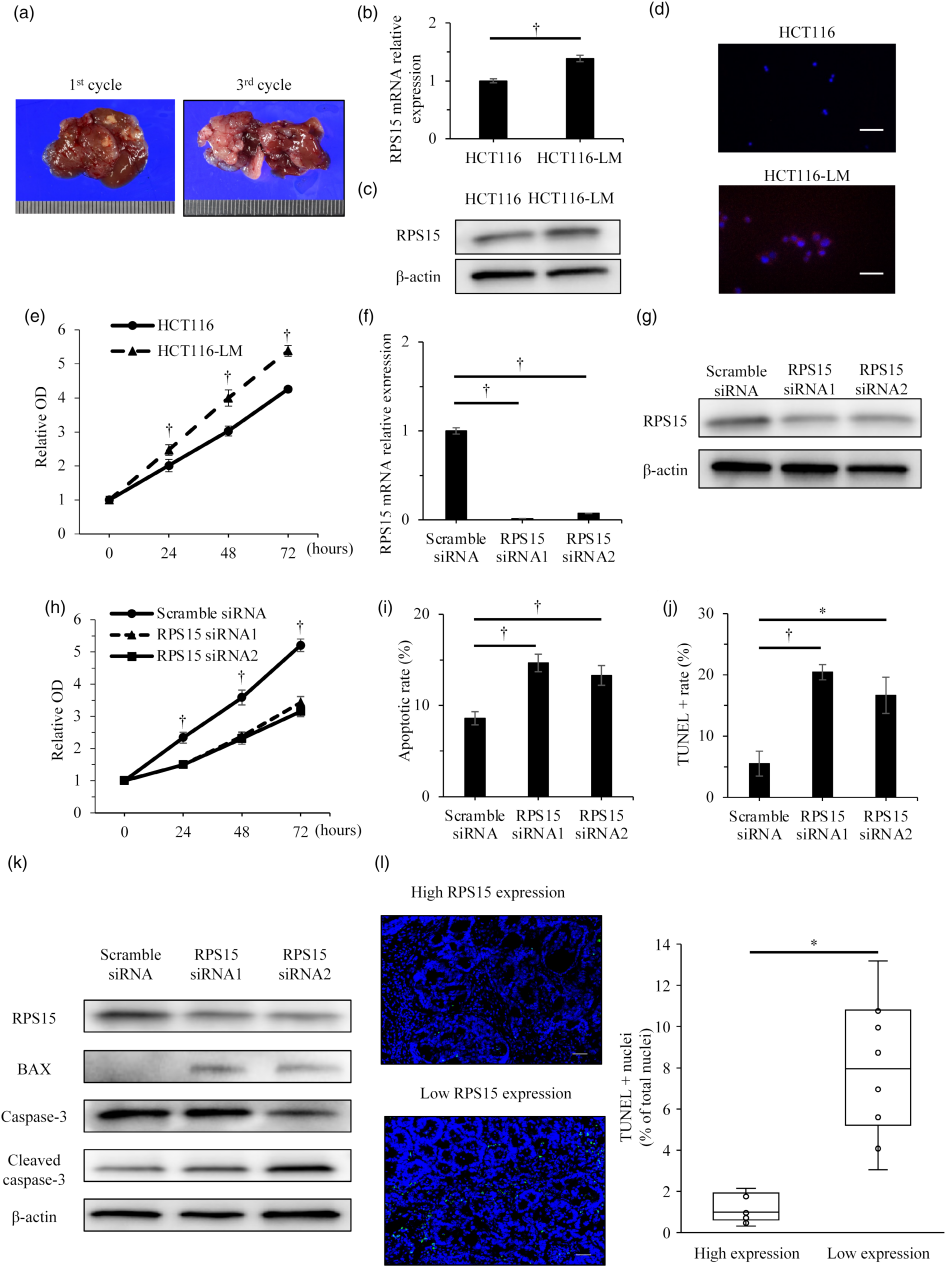
Knockdown of RPS15 expression in the highly liver‐metastatic cell line (HCT116‐LM) induced apoptosis in vitro. (a) The representative pictures of colorectal cancer liver metastasis (CRLM) tumors in the first and third cycle of in vivo selection. (b) Baseline RPS15 mRNA expression in HCT116 and HCT116‐LM cell lines. (c) Baseline RPS15 protein expression. (d) RPS15 expression and localization after in vivo selection by immunofluorescence. Nuclei were counterstained with 4′,6‐diamidino‐2‐phenylindole 9 (DAPI). Scale bar, 50 μm. (e) Proliferation of HCT116 cells and HCT116‐LM cells. (f) RPS15 mRNA expression in HCT116‐LM cells after transfection with RPS15 siRNA or scramble siRNA. (g) Expression of RPS15 protein after cell transfection with RPS15 siRNA or scramble siRNA. (h) Proliferation of HCT116‐LM cells after RPS15 knockdown. (i) Apoptosis of HCT116‐LM cells after RPS15 knockdown. (j) TUNEL‐positivity in HCT116‐LM cells after transfection with RPS15 siRNA or scramble siRNA. (k) RPS15 and apoptosis protein expression after RPS15 knockdown. (l) Quantification of TUNEL‐positive cells in CRLM specimens with high or low RPS15 expression. Scale bar, 50 μm. *p < .05, ^†^ *p* < .01.

The morphological appearance of the HCT116‐LM cells was similar to that of the parental line (HCT116). After three cycles of selection, the HCT116‐LM cells formed more metastatic nodules in the liver and showed significant increases in liver metastatic activity compared with HCT116 (Figure 4a). The two cell lines (HCT116 and HCT116‐LM) were examined for RPS15 expression by qRT‐PCR. RPS15 expression was higher in HCT116‐LM cells than in HCT116 cells (*p* = .0025, Figure 4b). Western blotting and immunocytochemical staining confirmed this result (Figure 4c,d), and HCT116‐LM cells showed significantly greater proliferative ability compared with HCT116 cells (*p* = .0039, Figure 4e).

### 3.6 Knockdown of RPS15 expression in the highly liver‐metastatic cell line (HCT116‐LM) by siRNA reduces its proliferative capacity and induces apoptosis in vitro

siRNA1‐ or siRNA2‐mediated knockdown significantly suppressed RPS15 expression to 7%–11% of the levels in the scrambled siRNA–transfected HCT116‐LM cell line (Figure 4f). Western blotting confirmed the suppression of RPS15 expression in the HCT116‐LM cell line (Figure 4g). Proliferation was significantly inhibited in the RPS15 siRNA–transfected cells to approximately 35%–40% of that seen in the scrambled siRNA–transfected cells (Figure 4h).

To investigate the molecular mechanism underlying the effect of suppressing RPS15 expression, annexin V assays were performed to detect any change in HCT116‐LM apoptosis. The knockdown of RPS15 induced an increase in apoptosis relative to the control level (Figure 4i). Furthermore, TUNEL assays showed that the percentage of TUNEL‐positive HCT116‐LM cells increased after RPS15 knockdown (Figure 4j). The silencing of RPS15 expression increased the expression of the apoptosis proteins Bax and cleaved caspase‐3 (Figure 4k). Thus, RPS15 expression could be involved in suppressing apoptosis in colon cancer cells, possibly affecting cancer progression.

### 3.7 Investigation of the relationship between RPS15 expression levels and apoptotis in CRLM specimens

To assess the relationship between RPS15 expression levels and apoptosis in CRLM, we performed the TUNEL assay in specimens from 20 CRLM cases, including 10 with high and 10 with low RPS15 expression. Low RPS15 expression was significantly correlated with the proportion of TUNEL‐positive cells among total cells, which varied by a factor of 6.7 (from 1.2% in the high‐RPS15‐expression group to 8.0% in the low‐RPS15‐expression group; Figure 4l).

## 4 DISCUSSION

In this study, for the first time, we determined that RPS15 is one of the DEGs specific to liver metastasis, and then demonstrated that high RPS15 expression in CRLM might be an independent and unfavorable prognostic factor using a public database and our cohort. In vitro, the inhibition of RPS15 reduced cell proliferation and induced apoptotic cell death by upregulating BAX. Furthermore, our established highly metastatic cell line, HCT116‐LM, had upregulated RPS15 expression similar to clinical specimens, and RPS15 inhibition decreased cell proliferation and increased the number of TUNEL‐positive apoptotic cells. Thus, RPS15 could be a promising target for the treatment of CRLM.

Hepatectomy is the only curative treatment for CRLM, but the recurrence rate is still high after hepatic resection.^15^ For colorectal adenocarcinoma, metastatic disease is the most common life‐threatening manifestation. The main metastatic mechanism of colorectal cancer is hematogenous dissemination, and the metastatic cascade theory explains the sequence of metastatic disease, which starts from the liver, goes to the lung, and then advances to other sites.^16^ Recurrence in the remnant liver after hepatectomy for CRLM may be explained by the hypothesized presence of micrometastases.^17^ Micrometastases cannot be detected by current imaging modalities, and the recurrence of many types of cancer is thought to be closely related to micrometastasis.^18^, ^19^, ^20^ In this study, the expression of RPS15 was upregulated in the specimens of liver metastases, and in the vivo experiment, RPS15 expression was upregulated in the highly metastatic colorectal cancer cell line compared with the primary colorectal cancer cell line. We established this cell line with high metastatic capacity after three cycles of in vivo tumor formation. It has been reported that specific genes are upregulated through the cycles of tumor formation, and that these genes are necessary for high metastatic capacity.^21^ The results suggest that targeting RPS15 might lead to the development of novel therapies to address postoperative micrometastasis.

RPS15 is a component of the 40S subunit, belongs to the S19P family of ribosomal proteins, and is essential for the final maturation and assembly of the 40S ribosomal subunit. Tumor cells are characterized by a higher production of ribosomes, which is necessary to sustain enhanced growth and subsequent cell division. Present in the cytoplasm, RPS15 has been found to be activated in a variety of tumors, including esophageal cancer, colorectal cancer, and insulinoma.^22^, ^23^, ^24^, ^25^ This study found that RPS15 expression was an independent poor prognostic factor in colorectal cancer liver metastases. Additionally, highly metastatic colorectal cancer cell lines exhibited increased expression of RPS15. Suppression of RPS15 expression decreased cell proliferative capacity and increased apoptosis, indicating that RPS15 might play a crucial role in metastasis and settlement of liver metastatic layers by controlling apoptosis‐related genes, including BAX, in CRC.

A recent report identified a truncating genetic variant in ribosomal protein S20 (RPS20), a novel colorectal cancer predisposition gene that encodes a component of the small ribosomal subunit (S20) in familial colorectal cancer type X (FCCX), which is characterized by hereditary nonpolyposis colorectal cancer without mismatch repair abnormalities.^26^ It has been suggested that it is involved in preribosomal RNA maturation and is responsible for normal cell proliferation.^27^ RPS20 has also been demonstrated to facilitate the proliferation and metastasis of renal cell carcinoma cells by stimulating the activity of AKT–mTOR and ERK–MAPK signaling pathways.^28^ RPS15, which we focused on, has been demonstrated to play a pivotal role in the organization of subunits distinct from RPS20, and, like RPS20, it has been shown to promote cell proliferation.^29^ In this study, we observed that suppression of RPS15 expression in colon cancer cells decreased cell proliferative capacity. Additionally, we found that RPS15 expression was increased in colon cancer cell lines with high liver metastatic potential compared to colon cancer cell lines. This suggests that RPS15, like RPS20, may contribute to the development of liver metastasis in colon cancer by promoting cell proliferation.

In this study, we analyzed DEGs from a public database of primary tumor, liver metastasis, and lung metastasis gene expression data and identified RPS15 as a liver metastasis–specific prognostic factor. The expression of RPS15 was assessed in both primary lesions and metastatic liver lesions of colorectal cancer. We demonstrated that high expression of RPS15 in the metastatic liver lesion but not in the primary lesion was a significant prognostic and recurrence factor (Figure 1f,g). The results indicate that RPS15 might be a useful marker following the formation of a metastatic lesion. This is because the clones of cancer cells with high RPS15 expression may survive and be enriched through the process of lesion formation. The seed and soil hypothesis proposed by Paget [30] suggests that the sites where metastases occur are defined not only by the tumor cell (seed) but also by the microenvironment of the secondary metastatic site (soil).^30^ This hypothesis posits that tumor cells exhibiting the metastatic phenotype represent a very small fraction of cells within the heterogeneous primary tumor. A heterogeneous primary tumor is postulated to give rise to a small proportion of tumor cells that gain the attributes necessary for metastasis over time.^31^ From this hypothesis, it is suggested that high RPS15 expression might be one of the attributes required for liver metastasis; further experiments would reveal the significance of RPS15.

In conclusion, RPS15 expression is an independent prognostic factor for patients with resected colorectal cancer liver metastases and represents a potential novel therapeutic target for patients with CLRM.

## AUTHOR CONTRIBUTIONS

Yoshihiro Sakano, Takehiro Noda, Shogo Kobayashi and Hidetoshi Eguchi designed the study. Yoshihiro Sakano, Daijiro Matoba, Takehiro Noda, Yoshito Tomimaru and Daisaku Yamada acquired the data. Yoshihiro Sakano, Takehiro Noda and Shogo Kobayashi interpreted the data. Yoshihiro Sakano, Takehiro Noda, Shogo Kobayashi, Hidenori Takahashi, Mamoru Uemura, Yuichiro Doki and Hidetoshi Eguchi drafted the manuscript.

## FUNDING INFORMATION

The authors received no specific funding for this study.

## CONFLICT OF INTEREST STATEMENT

The authors of this manuscript declare no conflicts of interest associated with this manuscript.

## Supporting information


Tables S1‐S2.
