# Supplementary material for: Clinical significance of ribosomal protein S15 expression in patients with colorectal cancer liver metastases
Source: J Hepatobiliary Pancreat Sci. 2024 Jun 4;31(9):611–24. doi: 10.1002/jhbp.12012 (PMC11503462; doi:10.1002/jhbp.12012)
Supplement: Supplementary file 1 — Tables S1‐S2. [file JHBP-31-611-s001.docx]

| Supplemental Table. 1 gene list | | |
| --- | --- | --- |
| gene symbol | Name | Summary |
| ACTR2 | actin related protein 2 | Unknown |
| AP2B1 | adaptor related protein complex 2 subunit beta 1 | The cytoplasmic face of coated vesicles in the plasma membrane. |
| BAD | BCL2 associated agonist of cell death | This protein positively regulates cell apoptosis. |
| BCAP31 | B cell receptor associated protein 31 | The anterograde transport of membrane proteins and caspase 8-mediated apoptosis. |
| CANX | calnexin | This protein facilitates protein folding and assembly. |
| CLTC | clathrin heavy chain | The intracellular trafficking of receptors and endocytosis of a variety of macromolecules. |
| CTNNA1 | catenin alpha 1 | The mechanoenzyme protein resulting in the reconfiguration of cadherin-actin filament connections. |
| GLUL | glutamate-ammonia ligase | This protein catalyzes the synthesis of glutamine from glutamate and ammonia in an ATP-dependent reaction. |
| GNB1 | G protein subunit beta 1 | G-protein synthesis protein. |
| GRN | granulin precursor | A family of secreted, glycosylated peptides. |
| HMGN1 | high mobility group nucleosome binding domain 1 | A protein that binds to nucleosomes DNA and is associated with transcriptionally active chromatin. |
| IK | IK cytokine | Unknown. |
| KDELR2 | KDEL endoplasmic reticulum protein retention receptor 2 | This protein is involved in the retention of soluble proteins resident in the lumen of the endoplasmic reticulum. |
| LAMC1 | laminin subunit gamma 1 | The major no collagenous constituent of basement membranes. |
| NFE2L1 | NFE2 like bZIP transcription factor 1 | This protein is involved in globin gene expression in erythrocytes. |
| NPM1 | nucleophosmin 1 | This protein is involved in several cellular processes, including centrosome duplication, protein chaperoning, and cell proliferation. |
| PRKAR1A | protein kinase cAMP-dependent type I regulatory subunit alpha | One of the cAMP regulatory subunits. |
| PTMA | prothymosin alpha | This protein is involved in negative regulation of apoptotic process. |
| PTPN21 | protein tyrosine phosphatase non-receptor type 21 | The protein is a member of the protein tyrosine phosphatase (PTP) family. |
| PTPRF | protein tyrosine phosphatase receptor type F | The protein is a member of the protein tyrosine phosphatase (PTP) family. |
| RAP1B | RAP1B, member of RAS oncogene family | This protein is a member of the RAS-like small GTP-binding protein superfamily. |
| RERE | arginine-glutamic acid dipeptide repeats | This protein colocalizes with a transcription factor in the nucleus, and its overexpression triggers apoptosis. |
| RPL6 | ribosomal protein L6 | This protein is a component of the 60S ribosomal subunit. |
| RPLP1 | ribosomal protein lateral stalk subunit P1 | This protein is a component of the 60S ribosomal subunit. |
| RPS15 | ribosomal protein S15 | This protein has an important role in component of the 40S ribosomal subunit. |
| RPS27 | ribosomal protein S27 | This protein is a component of the 40S ribosomal subunit. |
| RPS5 | ribosomal protein S5 | This protein is a component of the 40S ribosomal subunit. |
| SART3 | spliceosome associated factor 3, U4/U6 recycling protein | The protein is an RNA-binding nuclear protein that is a tumor-rejection antigen. |
| SPTBN1 | spectrin beta, non-erythrocytic 1 | Spectrin is an actin crosslinking and molecular scaffold protein that links the plasma membrane to the actin cytoskeleton. |
| SUMO3 | small ubiquitin like modifier 3 | This protein is covalently conjugated to other proteins via a post-translation modification known as simulation. |
| TAF10 | TATA-box binding protein associated factor 10 | This protein is involved in RNA polymerase II transcription. |
| THRA | thyroid hormone receptor alpha | The protein is a nuclear hormone receptor for triiodothyronine. |
| WDR1 | WD repeat domain 1 | This protein is involved in protein-protein interactions. |

| Supplemental Table. 2 Comparison of Patients' Characteristics | | | |
| --- | --- | --- | --- |
|  | High RPS15 expression (n=32) | Low RPS15 expression  (n=48) | *p-value* |
| Age (years) | 66 (41-82) | 65 (28-84) | 0.664 |
| Gender (male/female) | 21/11 | 33/15 | 0.770 |
| BMI (kg/m2) | 21.4 (17.4-34.9) | 21.5 (16.4-37.3) | 0.651 |
| Primary tumor location Colon/Rectum | 16/16 | 30/18 | 0.268 |
| Depth of invasion (T1/T2/T3/T4) | 0/4/24/4 | 0/6/34/8 | 0.872 |
| Lymph node metastasis (N0/N1/N2) | 11/13/8 | 20/23/5 | 0.230 |
| TNM Stage (Ⅰ/Ⅱ/Ⅲ/Ⅳ) | 1/5/8/18 | 2/11/11/24 | 0.859 |
| Primary histological type (well,mod/por) | 30/2 | 42/6 | 0.348 |
| Synchronous / Heterochronic | 18/14 | 24/24 | 0.583 |
| Neo Adjuvant Chemotherapy (Yes/No) | 17/15 | 26/22 | 0.927 |
| Child-Pugh classification (A/B) | 32/0 | 48/0 | 0.000 |
| CEA［ng/ml］ | 10.5 (1-423) | 5 (1-453) | 0.525 |
| CA19-9［U/ml］ | 41 (2-665) | 19 (0.4-766) | 0.141 |
| Number of liver metastasis (single/multiple) | 13/19 | 30/18 | 0.054 |
| Mean size of liver metastase［mm］ | 27.5 (10-70) | 26 (5-77) | 0.263 |
| Type of hepatectomy (partial/segmentectomy/sectionectomy/hemihepatectomy) | 19/6/5/2 | 39/6/1/2 | 0.081 |
| Recurrence (-/+) | 10/22 | 30/18 | 0.006 |
| Recurrence location (liver/other organ/both) | 10/7/5 | 6/6/6 | 0.263 |
| Death (-/+) | 20/12 | 35/13 | 0.325 |
| Cause of death (cancer-related/other) | 10/2 | 10/3 | 0.689 |
| Abbreviations: BMI, body mass index; CEA, carcinoembryonic antigen; CA19-9, carbohydrate antigen 19-9 | | | |
|  | | | |
